# TRAM (Transcriptome Mapper): database-driven creation and analysis of transcriptome maps from multiple sources

**DOI:** 10.1186/1471-2164-12-121

**Published:** 2011-02-18

**Authors:** Luca Lenzi, Federica Facchin, Francesco Piva, Matteo Giulietti, Maria Chiara Pelleri, Flavia Frabetti, Lorenza Vitale, Raffaella Casadei, Silvia Canaider, Stefania Bortoluzzi, Alessandro Coppe, Gian Antonio Danieli, Giovanni Principato, Sergio Ferrari, Pierluigi Strippoli

**Affiliations:** 1 Center for Research in Molecular Genetics "Fondazione CARISBO", Department of Histology, Embryology and Applied Biology, University of Bologna - Via Belmeloro, 8 - 40126 - Bologna, Italy; 2 Institute of Biology and Genetics, Marche Polytechnic University - Via Brecce Bianche, Monte D'Ago - 60131 - Ancona, Italy; 3 Department of Biology, University of Padova - Via G. Colombo, 3 - 35131 - Padova, Italy; 4 Department of Biomedical Sciences, University of Modena and Reggio Emilia - Via G. Campi, 287 - 41100 - Modena, Italy

## Abstract

**Background:**

Several tools have been developed to perform global gene expression profile data analysis, to search for specific chromosomal regions whose features meet defined criteria as well as to study neighbouring gene expression. However, most of these tools are tailored for a specific use in a particular context (e.g. they are species-specific, or limited to a particular data format) and they typically accept only gene lists as input.

**Results:**

TRAM (Transcriptome Mapper) is a new general tool that allows the simple generation and analysis of quantitative transcriptome maps, starting from any source listing gene expression values for a given gene set (e.g. expression microarrays), implemented as a relational database. It includes a parser able to assign univocal and updated gene symbols to gene identifiers from different data sources. Moreover, TRAM is able to perform intra-sample and inter-sample data normalization, including an original variant of quantile normalization (scaled quantile), useful to normalize data from platforms with highly different numbers of investigated genes. When in 'Map' mode, the software generates a quantitative representation of the transcriptome of a sample (or of a pool of samples) and identifies if segments of defined lengths are over/under-expressed compared to the desired threshold. When in 'Cluster' mode, the software searches for a set of over/under-expressed consecutive genes. Statistical significance for all results is calculated with respect to genes localized on the same chromosome or to all genome genes. Transcriptome maps, showing differential expression between two sample groups, relative to two different biological conditions, may be easily generated. We present the results of a biological model test, based on a meta-analysis comparison between a sample pool of human CD34+ hematopoietic progenitor cells and a sample pool of megakaryocytic cells. Biologically relevant chromosomal segments and gene clusters with differential expression during the differentiation toward megakaryocyte were identified.

**Conclusions:**

TRAM is designed to create, and statistically analyze, quantitative transcriptome maps, based on gene expression data from multiple sources. The release includes FileMaker Pro database management runtime application and it is freely available at http://apollo11.isto.unibo.it/software/, along with preconfigured implementations for mapping of human, mouse and zebrafish transcriptomes.

## Background

In the last few years it has became increasingly evident that, among the multiple gene expression regulation mechanisms, eukaryotic genes expression level is also dependent on their location within the genome [1]. For example, a more or less strong tendency for colocalization in the same chromosomal regions has been described for genes expressed at very high levels [2], genes constitutively expressed in most tissues (housekeeping genes) [3], genes encoding proteins assigned to the same functional pathway [4] or genes simultaneously expressed (coexpressed) in a particular tissue or organ [5]. The coexpression of colocalized genes could be determined by the conformation of chromatin domains to which they belong, or by local sharing of regulatory (e.g., enhancer) elements, thus raising questions about the functional significance of clustering of coexpressed genes [1]. Alternatively, clustering of genes could be explained by coinheritance, a selective pressure to maintain a genetic linkage among genes that encode for functionally related products and that will tend to be inherited together or, finally, it could merely reflect the origin of functionally related genes via tandem duplication of genes [6,7].

Further studies about the relationships between the expression of eukaryotic genes and their relative position in the genome are needed to clarify this biological issue. Such studies will take great advantage of the ever increasing amount of genomic-scale expression data obtained by serial analysis of gene expression (SAGE), gene expression microarrays or high-throughput RNA sequencing that are now made available in public databases. In fact, the transcriptome maps studies mentioned above showed the biological relevance of a global view of gene expression distribution by exploiting the availability of gene expression profile data obtained by the method of SAGE [2,3,5]. These studies contributed to challenge the traditional view that genes are randomly distributed along each chromosome in eukaryotic genomes. However, no computational biology tool for the generation and analysis of transcriptome maps was released to perform the algorithms described in these papers, with the exception of the web-based application "Transcriptome Map" [2,8]. Nevertheless, this only supports a limited number of input data types (derived from a few species, and, for human, only derived from SAGE experiments or from three Affymetrix microchip platforms), normalization methods and visualization options. The application "Caryoscope" [9] is a Java-based program, able to generate a graphical representation of microarray data in a genomic context. However, it is not intended to process input data (that must come from one single source, already containing all localization information for each element), or to perform any test of statistical significance on the resulting plot. The lack of software dedicated to constructing and analyzing transcriptome maps was already pointed out in 2006 [10], emphasizing that up until then, only algorithms or scripts had been presented and these were often tailored to specific uses (e.g., the study of a particular organism or the analysis of data derived from a single type of experimental platform). In addition, the tools that are available typically accept only gene lists as input and are not able to represent and analyze the continuous change, along the chromosome, of expression intensity assigned to overlapping regions of desired size, on the basis of the mean expression value calculated across all genes located in that region. This representation better reflects the biological reality of the quantitative changes of regional gene activity, rather than a simple count of the enrichment in differentially expressed genes, which is however also a desirable additional parameter of analysis.

**Figure 1:**
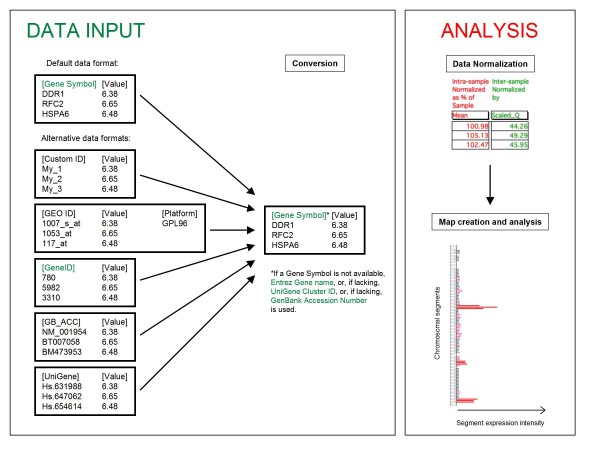
**General architecture of TRAM software**. The user is guided step-by-step through import and analysis of any gene expression profile dataset in text format. The gene identifiers of any type are converted in official gene symbols/gene names, followed by intra-sample as well as inter-sample normalization of gene expression values. The expression is mapped along each chromosome and graphically displayed on the basis of mean value for all genes included in each segment of arbitrary length. Over- and under-expressed regions are determined following statistical analysis.

These considerations underline the need for a general tool able to generate and analyze quantitative transcriptome maps from any source, provided that gene expression values for a certain gene set are available. Such a tool should also be capable of accepting and integrating data from multiple sources and be easily configurable for the investigation of any organism. Here we describe TRAM (Transcriptome Mapper), a user-friendly graphical interface software that may be run locally on personal computers (based on both Macintosh and Windows operating systems) and that meets and exceeds these specifications, by integrating original methods for parsing, normalizing, mapping and statistically analyzing expression data (Figure 1); in addition, it has the ability to easily generate maps showing differential expression between two sample groups, relative to two different biological conditions. The results of a test, using the hematopoietic progenitors CD34+ cells differentiation toward megakaryocytic cells (the large bone marrow cells whose fragments form platelets that are released into the blood) as a biological model, are also presented and discussed. This shows the ability of TRAM to identify chromosomal segments and gene clusters which are biologically relevant for the cell differentiation toward the megakaryocyte phenotype.

## Results

### Expression data import, parsing and normalization

The software is composed of a set of 37 related database tables, with 118 relationships among them. Some tables are designed to convert gene identifiers associated with expression data, e.g. GenBank accession numbers and/or UniGene cluster identifiers, into official gene symbols. Gene identifier conversion tables may be loaded or updated by the user, or are provided pre-loaded for human, mouse and zebrafish organisms. In addition, the user may download genomic data from Entrez Gene (e.g. chromosome number, chromosome lengths and genomic coordinates for known mRNAs) relating to the organism investigated. These data files are then easily imported and processed by the software during the set up process. Three species-specific pre-loaded (pre-setup) versions are also distributed.

In addition, TRAM makes fully original specific data management and analysis tools available, including a parser able to find the best and updated gene/RNA cluster name available to be assigned to a probe identifier. This is based on a converter of any RNA sequence accession number to the relative gene symbol, by searching an embedded parsed full UniGene updatable database table. For the human version, the parser locally resolves all 6,956,798 RNA sequence accession numbers, which are related to both known transcripts and to expression sequence tags (ESTs) included in *H. sapiens *UniGene build #228, December 2010. This allows a better conversion of those sequence accession numbers listed in a platform that may have been registered in the past few years without the availability of the presently corresponding gene symbols. For example, in the commonly used GPL96 GEO platform (Affymetrix HG-U133A microarray), 597 probe identifiers, with unavailable gene symbols in the Affymetrix platform scheme, were successfully assigned to mapped gene symbols or UniGene clusters.

A sample is defined as a homogeneous series of gene identifiers and their corresponding expression values, i.e. a list of values obtained in a single channel following a microarray hybridization experiment. To allow the comparison of expression data obtained from different samples, the absolute values of a sample may be converted into percentages of the mean (or median, or maximum) of all expression values within that sample. The software is also designed for the comparison between a sample (or a pool of samples) named 'A' and another sample (or a pool of samples) named 'B', each collected into specific tables. In this case, the ratio of 'A' and 'B' (named 'A/B') expression for each locus will be analyzed.

Although TRAM is a map-centred transcriptome analysis tool it can also summarize and allow the analysis of gene expression data of unmapped genes, exploiting its capability of parsing and normalization in order to highlight differential expression of single genes between two biological conditions even in the absence of data about genomic location of the gene.

### Scaled quantile normalization

We compared the correlation between sample datasets of the same pool but derived from different platforms, using the intra-sample normalization 'Mean' method (by which each value is expressed as percentage of the mean gene expression value for that sample). Inter-sample scaled quantile normalization always gave analogous or better results compared to standard quantile normalization. For example, the correlation coefficient between two series of locus-matched values obtained by distinct Authors, using different microarray platforms (samples A1 and A3, respectively; see Table 1), was 0.23 in absence of any inter-sample normalization, 0.34 following standard quantile normalization for all values and 0.41 following scaled quantile method. In the case of B10 and B12 samples, the quantile method worsened the correlation coefficient from 0.85 to 0.73, while this remained stable using scaled quantile normalization (0.83).

**Table 1:** Samples selected for the biological model used to test TRAM software

TRAM ID	GEO ID	Sample	GEO Platform	Microarray	Spots	**Ref**.
**Pool 'A'**						

A1	GSM321577	Mk (BM) (n = pool)	GPL96	Affymetrix U133A	22,283	[19]

A2	GSM321578	Mk (BM) (n = pool)	"	"	"	"

A3	GSM112277	Mk (PB) (n = 1, rep. 1)	GPL887	Agilent 1A	22,575	[20]

A4	GSM112278	Mk (PB) (n = 1, rep. 2)	"	"	"	"

A5	GSM15648	Mk (BM) (n = 6)	GPL96	Affymetrix U133A	22,283	[21]

A6	GSM8649	Mk (BM) (n = 6)	"	"	"	"

A7	GSM88022	Mk (PB) (n = 1)	GPL887	Agilent 1A	22,575	[22]

A8	GSM88014	Mk (PB) (n = 1)	"	"	"	"

A9	GSM88034	Mk (PB) (n = 1)	"	"	"	"

**Pool 'B'**						

B1	GSM321567	CD34+ (BM) (n = pool)	GPL96	Affymetrix U133A	22,283	[19]

B2	GSM321568	CD34+ (BM) (n = pool)	"	"	"	"

B3	GSM321569	CD34+ (CB) (n = pool)	"	"	"	"

B4	GSM321570	CD34+ (CB) (n = pool)	"	"	"	"

B5	GSM321571	CD34+ (PB) (n = pool)	"	"	"	"

B6	GSM321572	CD34+ (PB) (n = pool)	"	"	"	"

B7	GSM76923	CD34+ (BM) (n = 5)	GPL96	Affymetrix U133A	22,283	[23]

B8	GSM76924	CD34+ (BM) (n = 5)	"	"	"	"

B9	GSM76925	CD34+ (BM) (n = 5)	"	"	"	"

B10	GSM307288	CD34+ (BM) (n = 6, rep. 1)	GPL7091	Agilent 22 k A	16,391	-

B11	GSM307289	CD34+ (BM) (n = 6, rep. 2)	"	"	"	-

B12	GSM88023	CD34+ (PB) (n = 1)	GPL887	Agilent 1A	22,575	[22]

B13	GSM88003	CD34+ (PB) (n = 1)	"	"	"	"

B14	GSM23407	CD34+ (BM) (n = 1)	GPL201	Affymetrix HG-Focus	8,793	[24]

B15	GSM23410	CD34+ (BM) (n = 1)	"	"	"	"

B16	GSM23411	CD34+ (BM) (n = 1)	"	"	"	"

B17	GSM23408	CD34+ (BM) (n = 1)	"	"	"	"

B18	GSM23409	CD34+ (BM) (n = 1)	"	"	"	"

B19	GSM23406	CD34+ (BM) (n = 1)	"	"	"	"

**Sample**: Mk, megakaryocytic/megakaryoblast cells, obtained by in vitro differentiation of CD34+ cells; CD34+, undifferentiated CD34+ cells; (BM), (CB) or (PB): CD34+ cells derived from bone marrow, cord blood or peripheral blood, respectively. n = number of subjects from which the sample was derived (in some cases, where n = pool, the exact number of subjects included in a pool was not available). rep. = biological replicate. **Microarray**: U133A: Affymetrix Human Genome U133A Array; 1A: Agilent-012097 Human 1A Microarray (V2) G4110B; 22 k A: Agilent Human oligo 22 k A; HG-Focus: Affymetrix Human HG-Focus Target Array. When not directly provided, expression value was calculated as the median intensity value of a microarray feature minus the median background value.

In addition, we determined the standard deviation (SD, expressed as percentage of the mean) of measurements from different samples for housekeeping loci such as beta actin (*ACTB*) and a set of ribosomal proteins, using the intra-sample normalization 'Mean' method. In the absence of any inter-sample normalization, the SD for *ACTB *was 84.95 for pool 'A' (26 data points) and 148.80 for pool 'B' (60 data points). After applying quantile normalization to all available data, the SD became 35.36 and 78.91, and following the use of the scaled quantile method it changed into 51.44 and 54.75, for pool 'A' and 'B' respectively. Therefore, the scaled quantile method allowed both a decrease in the variability among the samples within a sample pool, and an increase in comparability between the 'A' and 'B' sample pools compared to a reference gene expected to be stably expressed in both pools. In the absence of any inter-sample normalization the SD for genes encoding small ribosomal proteins was 85.80 for pool 'A' and 138.75 for pool 'B' (mean of SD for 34 loci with "RPS" prefix, total data points were 402 for pool 'A' and 830 for pool 'B'). Following quantile normalization of all available data the SD changed to 66.01 and 78.25, and after using the scaled quantile method the SD decreased to 56.21 and 49.06, for pool 'A' and 'B' respectively, thus improving homogeneity within each sample pool as well as between the two sample pools.

### Generation and analysis of transcriptome maps

Two main types of analysis are available within TRAM: 'Transcriptome map - search for over/under-expressed segments' ('Map') mode and 'Search for clusters of neighbouring over/under-expressed genes' ('Cluster') mode.

**Figure 2:**
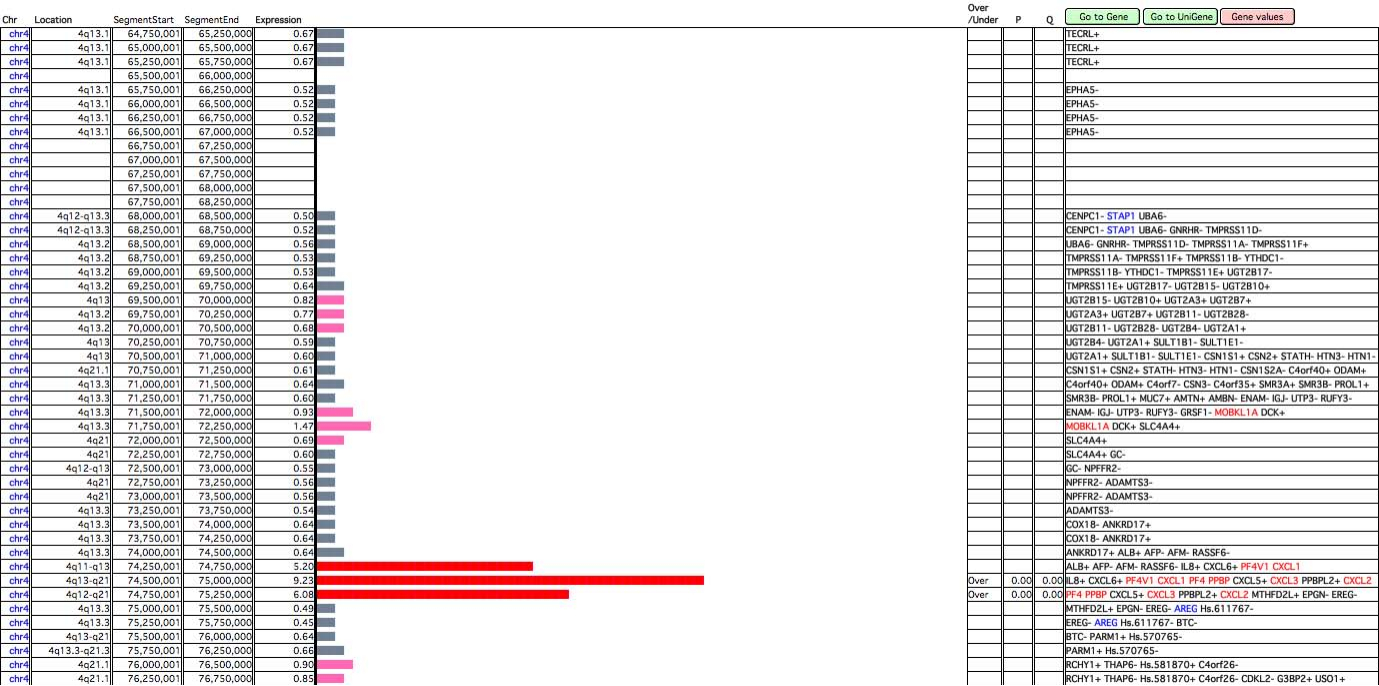
**Screenshot of the 'Map' graphical display of TRAM software (detail)**. The length of each horizontal bar is proportional to the mean gene expression within a chromosomal segment of 0.5 Mb. Consecutive bars are shifted by 250 kb. The vertical line represents human chromosome 4, from position 64,750,001 (start of the top segment) to position 76,750,000 (end of the bottom segment). The expression value on the left of each bar is derived from the analysis of the test set used (Table 1). Bars are colour-coded in proportion to their expression values. Segments, whose expression value is greater (or lower) than the chosen percentile threshold, are highlighted in the "Over/Under" field, which is only filled when they also include the user-defined minimum number of over/under-expressed genes that must be present in the segment. Statistical significance p- and q-values are calculated for these regions.

In 'Map' mode, the software generates a graphical map of the transcriptome showing a vertical line representing each chromosome. An expression value for a selected area of a chromosome is calculated as the mean for all available expression data relating to the genes included in that segment. The mean expression level of the segment is represented by an horizontal bar next to the corresponding segment of the chromosome, the bar size being proportional to the segment expression level (Figure 2).

Bars representing expression values included within the highest/lowest (n) user-defined percent of all segment expression values are pinpointed, thus highlighting genomic regions globally over/under-expressed with respect to the desired threshold. To avoid artefacts due to very high or very low expression of single genes in the region, the minimum number of over/under-expressed genes that must be present in the segment can be defined. The threshold for a gene to be considered as over- or under-expressed is the inclusion of the gene expression value within the highest/lowest (n) user-defined percent of all gene expression values.

The user may also set the 'Shift' parameter that causes the window to slide along the chromosome at pre-arranged intervals. In this way the user obtains a set of partially overlapping segments thereby attaining better sensitivity because a rigid division in chromosome segments, always starting from the fixed position 1, may let neighbouring over- or under-expressed genes be assigned to different segments.

The user maintains full control of the numerical data associated with each segment and may easily navigate among the map of the genes and gene expression values data tables. Segments may be explored and searched according to any desired criteria and sorted and processed like ordinary database records. Differential transcriptome maps, based on the ratio between corresponding gene expression values from two 'A' and 'B' samples or sample pools, relative to two different biological conditions, may be easily generated.

The statistical significance of the over/under-expression of each segment fulfilling the criteria to be tagged as over/under-expressed is displayed, and it is calculated as described in the "Statistical analysis" Methods section. The user may choose to refer the statistical calculations to data sets within each chromosome rather than to the whole genome dataset, in order to retrieve domains regionally over- or under-expressed within each DNA molecule. Differences between the genome- and chromosome-centred types of analysis are graphically highlighted when both have been performed.

### Search for clusters of neighbouring over/under-expressed genes

**Figure 3:**
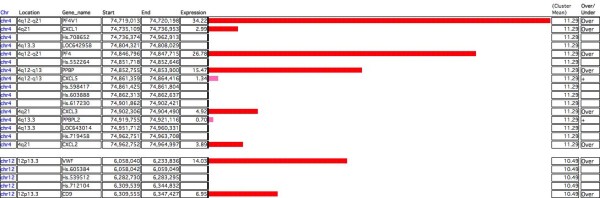
**Screenshot of the 'Cluster' graphical display of TRAM software (detail)**. Two example clusters, identified by default analysis of the biological model (Table 1) described in the text, are shown. The length of each horizontal bar is proportional to the mean 'A'/'B' ratio gene expression across all samples. Bar red colour indicates gene over-expression according to set criteria. Genes without associated expression values in the samples are shown but are not considered in the cluster construction. 'Gap' parameter was set equal to 1, so a maximum of one not over-expressed gene (hot pink colour bar) may separate two consecutive over-expressed genes. The cluster mean expression value, derived from all genes included in each cluster, is shown. The number of data points from which each value was derived, p-, q-value and length for each over/under-expressed cluster are also calculated (not shown here).

In 'Cluster' mode, the software searches for a set of at least two successive genes, arranged according to the position indicated by their known transcription start site, and over/under-expressed in terms of inclusion of the gene expression value within the highest/lowest (n) user-defined percent of gene expression values. In this type of analysis, each horizontal bar represents the expression level of an individual gene (Figure 3). Gene clusters are then built starting from over/under-expressed individual loci, if other contiguous genes fulfil the criteria defined for inclusion in the cluster. This analysis is complementary to that performed in 'Map' mode, which requires an arbitrary segment window.

The results of the analysis are displayed in the 'Cluster' layouts, as clusters of genes over/under-expressed. Each gene is actually a record (row) of the database. The user can find and sort genes and gene clusters using any desired criteria. Specific buttons help to retrieve entries from online databases for the desired genes. Clusters of differentially expressed genes between two different biological conditions may be easily generated, based on the ratio between corresponding gene expression values from two defined 'A' and 'B' pools.

Statistical significance of the over/under-expression of each gene cluster fulfilling the criteria to be tagged as over/under-expressed is displayed, and it is calculated as described in the "Statistical analysis" Methods section. In 'Cluster' mode the user may choose if statistical calculations are to be performed separately for each chromosome as it is possible in 'Map' mode. Moreover, the user may choose how many genes can be tolerated in the cluster between each gene pair counted in the cluster, even if they do not fulfil the user-set criteria.

### Biological model - Chromosomal segments

We compared several options of the software in the analysis of differential expression of pool 'A' (9 megakaryocyte cells (Mk) samples, including RNA from at least 21 different subjects) versus pool 'B' (19 CD34+ cells samples, including RNA from at least 41 different subjects) (Table 1). A total of 180,365 data points (gene expression values) from the pool 'A' and 294,987 data points from the pool 'B', relative to 17,676 distinct loci, for which an 'A'/'B' ratio value was determinable, were included in the analysis. Results obtained by default analysis (according to the parameters described in the "Methods" section) included 18 significantly over- or under-expressed segments ('Map' mode, Table 2) and 73 clusters ('Cluster' mode, Table 3). The use of inter-sample normalization (scaled quantile method) improved the identification of significantly over/under-expressed genome segments versus absence of any inter-sample normalization (18 vs. 12). In addition, segments enriched in relevant genes known to be over- or under-expressed in megakaryocytes/platelets were not identified in the absence of inter-sample normalization. For example an over-expressed segment on chromosome 17 was found to contain, among others genes, *ICAM2 *and *PECAM1*, as well as an under-expressed segment on 6p21.3 containing several HLA (human leukocyte antigen) system class II members. The differential expression of these genes is expected in the differentiation process studied in our model: *ICAM2 *is a functional integrin ligand present on platelets surface [11] and *PECAM1 *encodes platelet/endothelial cell adhesion molecule, while the down-regulated genes *HLA-DRA*, *HLA-DRB1 *and *HLA-DQA1 *are typically expressed in antigen presenting cells.

**Table 2:** Genomic segments significantly over/under-expressed in Mk cells (pool 'A') vs. CD34+ cells (pool 'B')

Chr and Location	Segment Start	Segment End	'A'/'B' Ratio	q-value	Genes in the segment
chr2 2q23-q24	160,250,001	160,750,000	0.398	0.00024	***BAZ2B- ****MARCH7- ****CD302- LY75-***

chr4 4q11-q12	57,500,001	58,000,000	0.421	0.00039	***HOPX- SPINK2- ****REST- C4orf14- POLR2B+ ****IGFBP7-***

chr4 4p15.32	15,500,001	16,000,000	0.427	0.00039	*FBXL5- ****BST1- CD38-**** FGFBP1- FGFBP2- ****PROM1-***

chr6 6p21.3	32,250,001	32,750,000	0.434	0.00079	*C6orf10+ BTNL2- ****HLADRA- HLADRB1- HLADQA1- ****HLADQB1- HLADQA2- HLADQB2-*

chrX Xp11.23	47,000,001	47,500,000	1.806	0.00505	*NDUFB11+ RBM10+ UBA1+ ****INE1+ ****USP11- ZNF157+ ZNF41+ ARAF+ ****SYN1+ TIMP1+ ****CFP+ ELK1+*

chr11 11q12.2	61,250,001	61,750,000	1.859	0.00573	*C11orf66+ SYT7- DAGLA- C11orf9+ C11orf10+ FEN1+ ****FADS1+ FADS2+ ****FADS3- RAB3IL1+ BEST1- ****FTH1+***

chr16 16p12.1	28,500,001	29,000,000	1.877	0.00248	*CLN3+ APOB48R+ IL27+ ****NUPR1+ ****CCDC101- SULT1A2+ ****SULT1A1+ ****EIF3C- ATXN2L+ TUFM+ SH2B1- ****ATP2A1+ ****RABEP2- CD19- NFATC2IP+ SPNS1+ ****LAT+***

chr17 17q23	62,000,001	62,500,000	1.957	0.00381	***CD79B- ****SCN4A- ****C17orf72+ ICAM2+**** ERN1+ TEX2+ ****PECAM1+ ****C17orf60- POLG2+ DDX5+*

chr6 6p21-1	43,500,001	44,000,000	2.196	0.00235	*XPO5- ****POLH+ GTPBP2+ MAD2L1BP+ ****MRPS18A+ VEGFA+ C6orf223+*

chr5 5qter	178,750,001	179,250,000	2.367	0.00222	*ADAMTS2- ****RUFY1+ ****HNRNPH1+ CANX+ MAML1+ ****LTC4S+ MGAT4B+ ****SQSTM1+*

chr11 11p15	5,000,001	5,500,000	2.384	0.00796	*MMP26- OR51L1+ OR52J3+ OR52A1+ HBB+ ****HBD+ Hs.20205+ HBG1+**** HBG2+ HBE1+ OR51B4+ OR51B2- OR51B6+ OR51M1- OR51I1+ OR51I2+*

chr17 17p13.2	4,750,001	5,250,000	2.620	0.00796	*MINK1+ CHRNE+ ****GP1BA+ ****SLC25A11+ RNF167+ ****PFN1+ ****ENO3+ SPAG7+ CAMTA2+ ****KIF1C+ ****GPR172B+ ZFP3- ZNF232- USP6+ ZNF594- RABEP1-*

chr4 4q13-q21	74,500,001	75,000,000	9.226	0.00000	*IL8+ CXCL6+ ****PF4V1+ CXCL1+ PF4+ PPBP+ ****CXCL5+ ****CXCL3+ ****PPBPL2+ ****CXCL2+***

Chr: chromosome; Segment Start/End: chromosomal coordinates for each segment. Bold and '+': over-expressed gene; bold and '-': under-expressed gene; '+' or '-': gene expression value higher or lower than the median value, respectively. Analysis was performed using default parameters (see "Methods" section). Segments are sorted by increasing 'A'/'B' ratio. In the 'Map' mode, TRAM displays UniGene EST clusters (with the prefix "Hs." in the case of *H. sapiens*) only if they have an expression value. Five out of a total of 18 segments are not shown for simplicity, because their over-expressed genes are overlapping with those highlighted in the listed regions. The complete results for this model are available along with TRAM software.

**Table 3:** Clusters of genes significantly over/under-expressed in Mk cells (pool 'A') vs. CD34+ cells (pool 'B')

Chr and Location	Cluster Start	Cluster End (Cluster size)	'A'/'B' Ratio	*q*-value	Genes in the cluster
chr19 19p13.3	827,831	856,246 (28,416)	0.103	0.00011	***AZU1- PRTN3- ELANE-***

chr4 4q11-q12	57,514,154	57,687,893 (173,740)	0.140	0.00084	***HOPX- ****Hs.630172 Hs.121443 Hs.673386 Hs.613041 Hs.677243 Hs.601479 Hs.44210 Hs.566128 ****SPINK2-***

chr10 10q23-q24	97,951,455	98,098,321 (146,867)	0.172	0.00084	***BLNK- ****Hs.716018 Hs.673979 Hs.444049 Hs.688648 Hs.679276 ****DNTT-***

chr1 1p36	26,644,411	26,647,014 (2,604)	0.192	0.00084	***CD52- Hs.597423-***

chr1 1q21	153,330,330	153,363,549 (33,220)	0.212	0.00011	***S100A9- S100A12- ****LOC645900**** S100A8-***

Chr6 6p21.3	32,407,647	32,611,429 (203,783)	0.217	0.00011	***HLADRA- ****LOC10028939 Hs.601001 Hs.544645 Hs.693189 Hs.654238 HLADRB5 Hs.664382 Hs.611927 Hs.606311 ****HLADRB1- ****Hs.706474 Hs.625753 Hs.691818 ****HLADQA-***

chr8 8p23.1	6,835,171	6,875,816 (40,646)	0.233	0.00084	***DEFA1- ****DEFA1B ****DEFA3-***

chr2 2p22.3	32,853,129	33,624,576 (771,448)	6.738	0.00084	***TTC27+ ****Hs.616001 Hs.664352 Hs.639709 Hs.683829 Hs.678473 Hs.623026 LOC285045 LOC100271832 ****LTBP1+***

chr11 11q12.2-q13.1	61,567,097	61,634,825 (67,729)	7.059	0.00084	***FADS1+ ****Hs.651782 Hs.621796 LOC100131326 Hs.667454**** FADS2+***

chr17 17q21.32	42,422,491	42,466,873 (44,383)	7.976	0.00084	***GRN+ ****Hs.602870 FAM171A2 LOC390800 ****ITGA2B+***

chr12 12p13.31	7,966,397	8,088,892 (158,906)	8.033	0.00084	***SLC2A14+ ****Hs.664258 Hs.539507 Hs.668117 LOC100130582 Hs.662096 ****SLC2A3+***

chr11 11p15.5	5,254,059	5,271,087 (21,857)	8.722	0.00010	***HBD+ Hs.20205+ ****Hs.295459 Hs.702082 Hs.702189 ****HBG1+***

chr12 12p13.3	6,058,040	6,347,427 (289,388)	10.492	0.00084	***VWF+ ****Hs.605384 Hs.539512 Hs.712104 ****CD9+***

chr4 4q12-q21	74,719,013	74,964,997 (245,985)	11.289	0.00000	***PF4V1+ CXCL1+ ****Hs.708652 LOC642958 ****PF4+ ****Hs.552264 ****PPBP+ ****CXCL5+ Hs.598417 Hs.603888 Hs.617230 ****CXCL3+ ****PPBPL2+ LOC643014 Hs.719458 ****CXCL2+***

Chr: chromosome; Cluster Start/End: chromosomal coordinates for each gene cluster. Bold and '+': over-expressed gene; bold and '-': under-expressed gene; '+' or '-': gene expression value higher or lower than the median value, respectively; gene name without '+' or '-' symbols: no expression value available in the investigated dataset. Analysis was performed using default parameters (see "Methods" section), choosing to list all colocalized known genes and mapped EST clusters, regardless of the availability of expression values for them in the investigated samples. Clusters are sorted by increasing 'A'/'B' ratio. Only the 7 cluster with the highest and the 7 cluster with the lowest cluster mean gene expression are shown (out of a total of 31 significantly over- and 42 significantly under-expressed identified clusters). The complete results for this model are available along with TRAM software.

The higher expression ratio between Mk and CD34+ cells (9.23) was observed in the segment at coordinates 74,500,001-75,000,000 on chromosome 4 (4q13-q21). All 10 genes in this location showed expression values greater than the median, and 6 out of 10 showed values within the higher 2.5th percentile. While it was known that several genes in this region are sequence-related and form a structural cluster of members of the CXC chemokine gene family (*CXCL1*, *CXCL5*, *CXCL3*, *CXCL2*), this finding highlights the simultaneous very high activity of genes such as *PF4V1 *(platelet factor 4 variant 1), *PF4 *(platelet factor 4) and *PPBP *[official name: pro-platelet basic protein (chemokine (C-X-C motif) ligand 7)], previously known as beta-thromboglobulin [12], following differentiation of CD34+ cells in megakaryocytes. The second segment with highest expression was located on chromosome 6 and contained over-expressed genes such as *GP1BA *[glycoprotein Ib (platelet), alpha polypeptide], encoding the alpha chain of megakaryocyte- and platelet-specific surface membrane Glycoprotein Ib, and *KIF1C *(kinesin family member 1C). Over-expression of kinesin 1C, which is recruited in neural cells APP (amyloid precursor protein) transport vesicles, was not to date described in Mk cells. However, it is known that during the complex and poorly understood process by which Mks generate platelets, kinesin motors carry platelet-specific granules and organelles over microtubules into the pro-platelets [13]. The third over-expressed segment spans the cluster of haemoglobins on chromosome 11, highlighting the known common early origin of erythroid and Mk cells [14]. Under-expressed segments included genes encoding surface antigens whose expression is known to be restricted to leukocytes or leukocyte subpopulations (e.g., CD302, LY75/CD205, CD38 and HLA-DR; Table 2), highlighting their down-regulation during differentiation of common CD34+ progenitors to megakaryocyte.

Switching from 'Mean' to 'Median' intra-sample normalization (values expressed as a percentage of the median value for each array) globally decreased sensitivity from 18 significantly over- or under-expressed genome segments to 10. However, one segment was identified only with the 'Median' mode. This segment includes *MYOM1 *(myomesin 1/skelemin), *MYL12A *(myosin, light chain 12A, regulatory, non-sarcomeric) and *MYL12B *(myosin, light chain 12B, regulatory) and it went undetected with the 'Mean' mode. Interestingly, myomesin 1 [15] and myosins [16] pathways have been involved in pro-platelet formation and platelet function. Some differences in the results while using different intra-sample normalization parameters are expected on the basis of the different distribution of the values for each sample. For example the sample mean value is greater than the sample median value if there is a tail of high values. Normalizing by median could uncover additional significantly over-expressed regions that were masked by the mean-based normalization.

Concerning inter-sample normalization, the scaled quantile adjustment appeared to increase sensitivity in the identification of significantly over/under-expressed segments with respect to the standard quantile method (18 vs. 13). In particular, the quantile method was not able to detect any under-expressed segment, such as that containing the HLA class II genes typical of leukocytes that were identified by scaled quantile adjustment.

Lowering or raising the threshold to define a gene and/or a segment as over/under-expressed makes the analysis more stringent or less stringent, respectively. We found that a good starting point is to use lower and upper 2.5th percentile, so that 5% of the data are included as positive results, which in a Gaussian distribution would roughly correspond to the percentage of values exceeding the mean by two standard deviations. The statistical test will compensate for the high number of segments or cluster marked as over/under-expressed obtained relaxing the threshold, by identifying which results are to be considered significant (Q < 0.05).

### Biological model - Gene clusters

In the 'Cluster' mode, the identified clusters included genes well known for being upregulated during megakaryocytopoiesis, as well as genes encoding leukocyte proteins expected to be down-regulated in the same context. For example, a cluster is composed by *DNTT*, encoding deoxynucleotidyl transferase, expressed in a restricted population of pre-B and pre-T lymphocytes, and *BLNK*, encoding B-cell linker, an adaptor protein that plays a critical role in B cell development (Table 3). In this mode of use, the physical contiguity of at least two over/under-expressed genes is considered as the bond for cluster definition rather than an enrichment of such genes in a genomic region independently of their order. Results are in part similar and in part complementary to those obtained in the 'Map' mode. In particular, the cluster with lowest 'A'/'B' ratio mean value turned out to be the series of genes *AZU1 *(azurocidin 1), *PRTN3 *(proteinase 3) and *ELANE *(elastase, neutrophil expressed), located on chromosome 19 and known to be coordinately expressed in a granulocyte-specific fashion [17], which resulted to be significantly under-expressed only in this mode of analysis. On the other hand, an over-expressed cluster composed only by the two contiguous genes, encoding platelet-specific proteins VWF (Von Willebrand Factor, present in the alpha-granules of platelets) and CD9 (a specific platelet marker), was identified on chromosome 12 (Table 3). This would have been undetected in the 'Map' mode because the minimal number of over/under-expressed genes required to be present in a chromosomal segment was by default set equal to 3. Another over-expressed cluster included *GATA1*, encoding a major transcription factor for megakaryocytopoiesis.

The modification of parameters used for 'Cluster' mode analysis, such as those above listed for the 'Map' mode, had analogous effects on the results.

Some additional significant results were obtained by setting chromosome-specific thresholds for the analysis, rather than using the whole genome gene set as a reference. For example, the cluster of the two genes *HIPK2 *(a nuclear kinase that interacts with homeodomain transcription factors, previously associated with megakaryocyte lineage) [18] and *TBXAS1 *[official name: thromboxane A synthase 1 (platelet), catalyzing the conversion of prostaglandin H2 to thromboxane A2, a potent vasoconstrictor and inducer of platelet aggregation] scored as significantly over-expressed on a local basis when the gene expression of chromosome 7 but not whole genome dataset was considered.

In both 'Map' and 'Cluster' modes, TRAM displays EST clusters, in addition to known genes, mapped in the region of interest. In the case of 'Cluster' mode this occurs independently of the availability of expression values for the loci within the cluster extension (Table 3).

The results for each mode of analysis of the presented test model, using default parameters, are available in the folder 'Biological_Model_Test' of the TRAM distribution, allowing the user to explore the model following variation of analysis parameters.

Individual gene expression values, summarized for each of the two pools 'A' and 'B', can be visualized and sorted in the 'Cluster' results layout. Amongst the first 12 genes with the highest 'A'/'B' ratio (*PF4V1*, *PF4*, *GP1BA*, *PPBP*, *RGS18*, *CMTM5*, *SLC44A1*, *VWF*, *SH3BP5*, *HSPC159*, *ITGA2B *and *HBG1*, ranging from 34.22 to 11.92 expression ratio, in this order, between CD34+ and Mk cells), 7 were placed in one significantly over-expressed segment or gene cluster by the transcriptome mapping analysis.

The *CD34 *gene was under-expressed in Mk cells compared to CD34+ cells, as expected (mean ratio across all samples was 0.29, within the lowest 2.5th percentile of 'A'/'B' ratios).

## Discussion

Here, we have described an original software named TRAM, designed to create and analyze transcriptome maps for any organism, based on gene expression data in a general form and able to generate a relational, fully-indexed map database, usable on Macintosh and Windows operating systems-based computers. The 'Map' mode allows the identification of chromosomal regions of defined size (with the possibility of using a sliding window) whose expression is defined as the gene expression average of the genes contained in the segment. This segment, also, must contain a specified number of loci transcribed beyond a desired threshold. The wide flexibility of the parameters required for the building of the Map (e.g., the independence of the threshold value chosen to consider each gene as over/under-expressed from the threshold value set to define a genomic segment as over/under-expressed) makes an open exploration of the expression data feasible at different levels; in addition, an estimate of statistical significance for the definition of a segment as over/under-expressed can then be obtained. This type of analysis considers the global expression of all genes in the region, regardless of their exact reciprocal position: in fact, it has been shown, for example for genes belonging to the same functional pathway, that clustering is loose and individual genes may be spread, despite remaining closer to each other than expected by chance [4]. In addition, we added a complementary mode of data visualization and analysis, the 'Cluster' mode, where the window width is defined by a number of clustered genes rather than nucleotide range. This method can consider the gene-by-gene order in the region and can provide results about clusters of over/under-expressed genes that are adjacent or separated by a small user-defined number of not over/under-expressed genes within the cluster. All TRAM data and results tables are widely interconnected by simple navigation buttons, as well as linked to the relevant entries available on line (via automatic opening of the default web browser).

The novelty of the tool is supported by several arguments. Firstly, the uniqueness of TRAM general basic architecture, which dynamically integrates an advanced and flexible relational database with parsing and meta-analysis capability, a map graphic displayer and a two-modes ('Map' and 'Cluster') analyzer searching for significantly over/under-expressed genomic regions, starting from any source of global gene expression profiles data (Figure 1). The TRAM data model is also unique in two other aspects: users are given direct access to expression numerical values, which are always visualized near the horizontal expression bar used to visualize the expression intensity of chromosomal segments or clustered genes localized on the map; moreover, the users do not need to provide genomic coordinates for the investigated genes, whose location is resolved by the pre-setup gene database table. A series of buttons allows an easy, transparent tracking of gene expression measurements from raw input data to normalized values, up to expression intensity display on the map.

In addition, TRAM makes original specific data management and analysis tools available such as: a parser which is able to find the best and updated gene/RNA cluster name suitable to be assigned to a probe identifier (e.g., the parser locally resolves all ~7 millions human RNA sequence accession numbers included in the latest available *H. sapiens *UniGene version); a novel effective method for the normalization of data derived from platforms with highly different number of probes (scaled quantile) which allows more samples to be included in a biologically homogeneous sample pool and maximizes gene expression information that may be extracted from each sample; the statistical analysis, based on individual chromosome data summary in addition to genome data summary, emphasizing local effects expected on the basis of the behaviour of single chromosomes with respect to chromatin organization and gene expression regulation.

Finally, a powerful feature of TRAM is its ability to compare, within the same analysis, the transcriptome maps derived from two datasets (or two pools of datasets) related to different biological conditions (indicated as 'A' and 'B'), such as two tissues or cell types, two developmental stages, normal vs pathological cells or cells maintained in absence or in presence of a substance. The generation of a transcriptome map of the relative 'A'/'B' ratio expression, allows the easy investigation of regional differential expression without the need to generate the results separately for the two datasets or pool of datasets and to devise additional calculations to compare them.

TRAM was able to generate original results of relevant biological interest in the *ab initio *modelling of differentiation from CD34+ stem cells to megakaryocyte (Mk) cells in a meta-analysis of a total of 28 publicly available microarray datasets obtained from different sources. Many genes with a fundamental role in Mk/platelet biology, known since early classical studies (see "Results" section), were shown to significantly colocalize in genome segments or in clusters of adjacent genes. Moreover, additional regions significantly over/under-expressed during megakaryocytopoiesis were identified (Table 2 and Table 3). These results are original compared to the data analysis presented in the relative primary works from which expression data were derived [19-24]. This may be ascribed to the lack of data integration in the original studies (analysis was typically applied only to the datasets produced in the context of the work itself) [19-24], to the lack of a search for local enrichment of over/under-expressed genes [20-24], to the use of a different analysis pipeline when a localization study was performed (in particular, use of gene lists as a starting point rather than the actual mean expression value of the genes in a region) [19], or to the different biological model considered (when the study was not intended to investigate differential expression during differentiation of CD34+ cells toward Mk cells) [21,23,24].

EST clusters can be mapped to the region of interest in addition to known genes by exploiting an original integration between NCBI UniGene and UCSC Genome Browser data (Table 2 and Table 3). This feature offers useful hints for the functional investigation of uncharacterized transcripts, on the basis of their presence, and in case of their over/under-expression, within genomic regions differentially expressed in a certain biological context.

**Figure 4:**
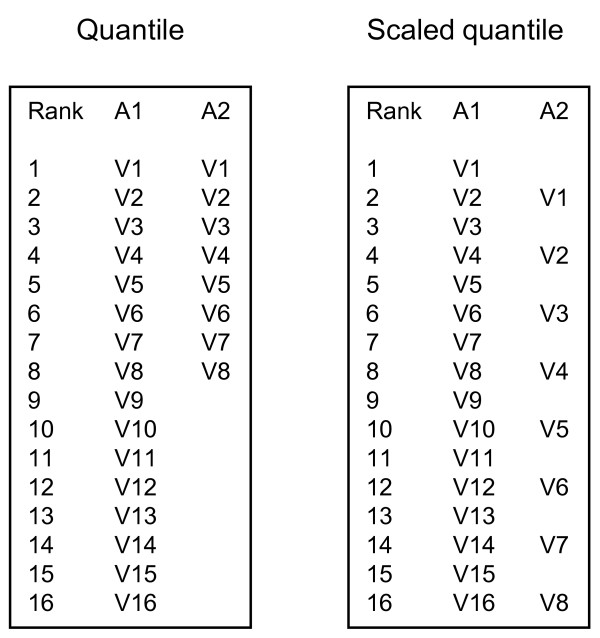
**Scaled quantile normalization: concept**. If two data columns with different numbers of values, derived from two A1 and A2 samples, respectively, are individually sorted by magnitude of expression to obtain the mean value for all values with the same rank, i.e. in the same row (quantile normalization), the highest values in the sample A2 will be aggregate to the intermediate values in the sample A1. Proportional scaling of A2 ranks aligns them to A1 values located in analogous ordered positions with respect to each sample whole distribution (scaled quantile inter-sample normalization), allowing low, intermediate and high values to be aggregated with suited corresponding values from the other sample(s).

While feasibility of integration of gene expression profile data, obtained from different experimental platforms or investigators, is highly desirable to build transcriptome maps representing all information available for a certain biological condition, the occurrence of systematic errors associated with each experimental situation requires advanced methods of inter-sample data normalization, such as the widely accepted quantile normalization [25]. However, this method may cause loss of data due to the removal of all genes whose expression values are missing for any dataset in order to obtain a fully filled data matrix, representing each sample as a column and the values for each gene as a row (for an example of this filtering see [26]). Alternatively, some Authors retain all data values in quantile normalization by placing missing values at the end of each sorted column [27]. In such cases, all available data are analyzed but with an artefact due to the misalignment of values included in similar classes of expression level, compared to their sample of origin (Figure 4). The original method of scaled quantile we propose here in order to properly manage data derived from platforms with different number of analyzed genes, has proven to be effective, allowing maximization of information that can be extracted from all pertinent available data.

Interestingly, after selecting for the analysis only samples homogeneous with respect to the used experimental platform, a decrease in sensitivity was observed, thus showing the effectiveness and usefulness of analysis starting from multiple sources. For example, by performing the analysis only on the datasets obtained with commonly used Affymetrix U133A microarray (GPL96 GEO platform) among those listed in Table 1, only 5 vs. 18 significantly over/under-expressed chromosomal segments, and 68 vs. 73 gene clusters were found, compared to the analysis integrating data from the whole available pool of samples.

A variation of analysis parameters allows the exploration of data from different points of view. The final statistical significance test will provide the actual reliability of the corresponding results independently of the parameters selected to define the thresholds for considering segments and genes as over/under-expressed (selection performed at the start of the data analysis by descriptive statistics). For example, lowering the threshold to consider segments and genes as over/under-expressed will retrieve a larger number of differentially expressed regions but this will be taken into account during the calculation of the statistical significance of each result where only a minor fraction of these regions will have a q-value (p-value corrected for FDR) < 0.05. It is noteworthy that, among the first 12 individual genes with the absolute highest 'A'/'B' ratio between CD34+ and Mk cells, 7 were placed in one significantly over-expressed segment or gene cluster by the transcriptome mapping analysis. In addition, valuable information may also be extracted by using the capability of TRAM to numerically describe the normalized and summarized intensity of transcription along each chromosome. In this way a user could readily search, find and sort regions with no positive expression values despite containing known genes ("expression deserts").

Taken together, the described features of TRAM make it difficult to compare in details this tool with all other tools that, to our knowledge, are described in the literature as being capable of transcriptome mapping and analysis. This is because TRAM is actually a suite of different and strictly integrated data parsing and displaying as well as analysis tools. In particular, the existing software reviewed in the "Background" section, accept gene lists as an input (lists are to be obtained through different dedicated tools), so they cannot represent and analyze the gene expression level changing along the chromosome. Neither will they generate differential expression maps comparing two different biological conditions such as the one we have discussed in our biological model showing progression from CD34+ cells to megakaryocytes. The local differential gene expression between two conditions is a function offered by the "R" library MACAT [28], whose Authors used a set of publicly available microarray data [29] related to human T- (n = 43) and B-cell (n = 205) paediatric acute lymphoblastic leukaemias as biological test example. Only one chromosomal region in chromosome 6 was found to be differentially expressed between T- and B-leukaemia cells. This was a biologically meaningful finding since HLA genes are localized in this region and they are known to be under-expressed in T-cells vs. B-cells [28]. TRAM was able to replicate this result by using default analysis parameters. In addition, TRAM was able to individually list the loci under-expressed in T-cells present in this region (6p21.3, coordinates 32,2500,000-33,000,000, q-value < 0.000002, under-expressed genes: *HLA-DRA*, *HLA-DRB1*, *HLA-DQA1*, *HLA-DQB1*, *TAP2 *- involved in antigen presentation -, *HLA-DMB*, *HLA-DMA*). Moreover, TRAM has been able to identify three additional differentially expressed chromosomal regions, each of which contained several genes over-expressed in T-cells, one on chromosome 1 (1q22-q23, q-value = 0.000007, genes: *CD1D*, *CD1A*, *CD1B*, *CD1E*) and two on chromosome 11 (11q12.2, q-value < 0.0007, genes: *CCDC86*, *GPR44*, a chemoattractant receptor homologous molecule expressed on T-helper type 2 cells, and *CD5*; 11q23, q-value < 0.0007, genes: *CD3E*, *CD3D *and *CD3G*). These regions are of remarkable biological and clinical interest because they contain clusters of genes related to CD1, CD5 and CD3, respectively; these are well known as main and universally used specific surface markers of T-cells. In the "Cluster" mode, TRAM identifies 35 gene clusters significantly over- (n = 16) or under-expressed (n = 19) in leukaemic T-cells compared to B-cells, including several other genes known to be T- or B-cell specific (data not shown). Finally, MACAT is limited to the analysis of Affymetrix microarray, further underlining the need for a tool open to all platforms as well as to cross-platform analysis.

The batch effects are the systematic differences between batches (groups) of samples in microarray experiments due to technical reasons, such as variability in materials, protocols or operators, possibly introducing a bias able to confound true biological differences (recently reviewed by Luo and coll. [30]). The TRAM data model described appears to be intrinsically resistant to the influence of batch effects, for the following reasons: the TRAM locus-centred data model does not attempt to separate subgroups within a sample pool, because it is assumed that the samples come from the same biological condition (e.g. cell type, disease) for which only one aggregate value per locus is obtained and considered; the TRAM algorithm is non-parametric at different levels of normalization and analysis and this is expected to reduce the noise due to different value scales; the biological model discussed above deliberately used data from different platforms, protocols and operators, showing results coherent with the current biological knowledge and even better with respect to the results obtained analyzing data deriving from a single platform. However, if the user suspects that batch effects could confound the results, in particular if two single and distinct batches of samples are loaded as pool 'A' and 'B', respectively, it could be useful to attempt removing batch effects from the raw data using one of the existing tools [30] prior to importing data in TRAM.

While this manuscript was being revised, two novel software were described in addition to those reviewed in the "Background" section, able to analyze local enrichment of over/under-expressed genes. CROC [31] also uses the hypergeometric distribution to find genomic regions or gene clusters enriched in over/under-expressed genes, and supports calculations based on both whole genome data and individual chromosome values. However, like the previously published REEF tool [10], it accepts gene name lists as an input and it is not designed to parse, normalize and map original expression data and to display the corresponding quantitative transcriptome maps. The Integrated Genome Browser [32] can load expression data and visualize them along an *x *axis representing the chromosome sequence. However, it lacks any function of data integration, normalization and analysis (Figure 1), being essentially a graphical display tool for expression data, like the previously published program ChromoViz [33].

The large agreement of TRAM results, obtained without any *a priori *specific assumptions, with classical biological knowledge about megakaryocytopoiesis, shows that TRAM can perform integrated analysis of expression data from multiple platforms producing high confidence lists of over/under-expressed chromosomal segments and clustered genes. In conjunction with our previous implementation of a GenBank format full parsing system [34] (currently undergoing complete redesigning within FileMaker Pro 7 architecture) and UniGene Tabulator [35], TRAM may also contribute to the building of a novel, relational, multi-purpose, user-friendly and modular platform for the large-scale integrated analysis of genomic and post-genomic data.

## Conclusions

We have here described a unique package able to create and analyze transcriptome maps by integrating gene expression profile data from multiple sources and generating a relational, fully-indexed database-structured map, usable on Macintosh as well as on Windows operating systems-based computers, features that are non commonly available in other applications.

TRAM provides a simple and intuitive system for the display and analysis of gene expression data within a single solution, including built-in multiple gene identifier conversion modules, intra-sample and inter-sample data normalization, map comparison between two biological conditions, graphical display and highly flexible data analysis (by both descriptive and inferential statistics) that has proven to generate results of biological interest.

The current release of TRAM software is freely available at TRAM home page [36]. We also distribute preconfigured implementations ready for analysis of *Homo sapiens *(human), *Mus musculus *(mouse) and *Danio rerio *(zebrafish) gene expression profiles.

## Methods

### Database development

TRAM was developed within the FileMaker Pro environment. This is a database management system with a user-friendly graphical interface usable on Macintosh and Windows operating systems-based computers. All data import, expression analysis and results of graphical output functions are obtained combining FileMaker Pro scripts and calculated fields (i.e. fields automatically calculating their result processing value from other fields by a pre-defined formula). No additional plug-in or software are required. A specific advantage of this platform as a transcriptome map generator and analyzer is the relational database environment at its core. As a consequence, each dataset in any table (e.g. gene expression values, gene names, expression value of chromosomal segments or gene clusters) is structured as a series of records that may be easily sought according to the desired criteria and then sorted, exported, and possibly related to other database tables. In this way, the graphical display of the map allows the user to maintain full control over the original expression data values at the basis of the map.

The freely distributed licensed runtime application allows full data import and export in several formats, as well as full record management and analysis script execution. Only for the creation of new fields, further calculation or additional relationship definition an original copy of FileMaker Pro version 10 (or higher) package is required.

### TRAM set up

In order to link gene identifiers to the corresponding gene symbols/gene names, it is possible to import in TRAM any text data file containing essential description (e.g., probe identifiers list and matching gene symbols or GenBank sequence accession numbers) for each experimental platform used to assess gene expression level in the examined samples (Figure 1). A typical use is loading a GEO [37] Platform file, in order to parse expression datasets obtained using that platform. Pre set-up human, mouse and zebrafish versions are distributed following loading of the most used GEO Platforms for those organisms. In order to uniform the assignment of gene identifiers to standard gene symbols, in absence of an available official gene symbol or an Entrez Gene [38] name, the UniGene [39] Cluster identifier (UniGene ID) is used as the gene name, if available, while the GenBank accession number is used, if provided, in absence of any match to an Entrez Gene or UniGene entry (Figure 1).

TRAM 1.0 distribution was set up using data available at January 2011, downloading from Entrez Gene the gene localization data and parsing from UniGene tables, allowing the conversion of any RNA or expression sequence tag (EST) GenBank accession number into the corresponding gene symbol [35]. In the case of human UniGene latest available version (build #228), about 7 millions RNA and EST code data were imported in TRAM.

In addition, localization of EST clusters, which are sequences not characterized as official genes but represented in the transcriptome, was derived from UCSC "ESTs" track in the UCSC Genome Browser [40], which is also imported and processed during the TRAM set-up. A relationship between UniGene and UCSC ESTs data allows to determine the minimal available start genomic coordinate and maximum available end genomic coordinate for each set of ESTs belonging to the same UniGene cluster. These coordinates are operatively considered the limits of the locus while constructing the transcriptome map. Clusters containing ESTs mapped on different chromosomes are not further considered in the building of the map, as well as those with ESTs mapping on very distant positions on the same chromosome. To this aim, we set a rather conservative limit of 250,000 bp for TRAM, considering that the Entrez Gene set of 27,018 human genes, that is the largest known, has a mean size of 46,210 bp and a standard deviation of 107,161 bp, therefore our limit is equivalent to considering a size range within mean plus or minus 2 SD (approximately 95% of values in a Gaussian distribution). This correction effectively removes approximately 3,000 transcripts, erroneously mapped to regions of several Mb or tens of Mb. The user retains the possibility to inspect the list of EST clusters with a genomic extension greater than 250 kb present in a given chromosome segment, even if they are not considered in the creation of the transcriptome map.

### Expression data import, parsing and normalization

Each series of data related to a TRAM 'Sample' is defined as a 'distinct biological sample'. For example, a sample should be a single channel in the case of two channels experiment, each channel data being imported as a distinct data file. The expression data file may be any tabulated (tab-delimited) text file containing two columns separated by a 'TAB' character (tabulator key, ASCII9): a gene identifier and a numerical expression value, respectively. Gene symbol, Entrez gene name, custom identifier, GEO Platform probe ID or GenBank accession number are accepted as gene identifiers: the first two by default, the others provided that the software has been appropriately set up.

The expression value is usually the pre-processed intensity value (i.e., the value assigned to the spot as it has been processed by the software of the specific experimental platform used, for example, following background subtraction for a microarray spot). An internal utility interactively assists the user in the preparation of text files in the required format, starting from raw expression data. Batch import of a large number of data files is possible. Each sample or set composed of any number of samples may be imported in one of two pools, 'A' or 'B', relative to two different biological conditions that may be then easily compared.

TRAM is able to perform some useful data normalization methods (Figure 1) to allow comparison of gene expression data obtained by different biological samples and/or by different experimental platforms.

Intra-sample (e.g., intra-array) normalization works within each distinct sample data, while inter-sample (e.g., inter-array) normalization is simultaneously applied to the desired sample set.

The user may select different combinations between these types of normalization.

Intra-sample normalization methods are 'Mean' or 'Median' (each value is expressed as the percentage of the corresponding sample mean or median value, respectively; this is equivalent to the classic "global normalization" in the microarray data analysis [41]) and 'Max' (each value is expressed as the percentage of the corresponding sample maximum value, equivalent to the classic "scale normalization"). These methods rescale values within each data set using a standard internal reference for each sample. Inter-sample normalization method is the commonly used "quantile" algorithm [25]. Implementation of this algorithm in the database structure at the core of TRAM is realized as follows: each intra-sample normalized value is given a rank following sample data sorting in ascendant order, then the mean value for all the values with the same rank across all samples is calculated. This mean value is assigned as the expression value to each gene with the same rank in each sample. An original variant of this method implemented in TRAM is described below. The inter-sample normalization methods rescale values across a whole sample set, allowing inter-sample comparison.

The summary of gene expression values, under the current mode of normalization, may be viewed as an indexed database table summarizing all data points available in the sample pool for each locus. The mean value of the data points available for each locus is considered the expression value for the respective gene and it is used in the subsequent analysis.

### Scaled quantile normalization

The quantile method assumes that each sample has the same number of values. However, datasets obtained from different platforms used to assess the gene expression profile, may have highly different numbers of values. In this case, applying the quantile method to the matrix resulting after aligning and sorting values from each sample (represented as a column) gives raise to artefacts, in that the highest values of a sample are summarized with intermediate values of samples with a greater number of values (Figure 4). To correct for this artefact, we applied the following formula in TRAM: Scaled Rank = Rank * Max Rank/Max Sample Rank, were 'Rank' is the rank (position in the ranking) of the value in the sample data column sorted in ascendant order, 'Max Rank' is the highest rank present in the whole sample dataset, and 'Max Sample Rank' is the highest rank assigned within each considered sample. The result of the calculation is rounded to the nearest integer number. The adjusted rank is then used to calculate the mean value across all genes which had the same rank assigned (Figure 4). In the case that the experimental platforms have the same number of features, the scaled quantile is identical to quantile. Comparability of values is best attained if previous intra-sample normalization has been performed too.

### Generation and analysis of transcriptome maps

In the 'Map' mode of analysis, TRAM will generate a graphical map of the transcriptome showing a vertical line representing each chromosome (Figure 2). An expression value is associated to each segment of the line, whose size is determined by a window (in bp) set by the user. A differentially coloured horizontal bar is displayed for each chromosomal segment, with a length proportional to the expression value assigned to the relative segment. This value is the mean for all available expression data related to genes included in each segment. The available settings for this analysis are: Window, which defines the length for a segment; Sliding window shift, which defines the overlapping region between a segment and the next one; Percent (segment), which defines the threshold required to consider a segment as over/under-expressed, in terms of inclusion of the segment expression value within the highest/lowest (n) percent of segment expression values; Percent (gene), which defines the threshold expression value to consider a gene as over/under-expressed, in terms of inclusion of the gene expression value within the highest/lowest (n) percent of gene expression values; Number of genes in the window, which defines the minimum number of over/under-expressed genes required to mark the segment with the tag of over/under-expressed, respectively.

The calculation of statistical significance of the over/under-expression of the segment is performed as described in the "Statistical analysis" Methods section below.

### Search for clusters of neighbouring over/under-expressed genes

In the 'Cluster' mode of analysis, TRAM will search for sets of at least two contiguous/neighbouring genes, all expressed beyond a defined 'n' threshold, i.e. with expression values higher than the (100 - 'n') percentile or lower than the 'n' percentile. In this mode, results are centred on individual differentially expressed loci without any bond about the length of the over/under-expressed region.

The available settings for this analysis are: Percent (gene), which defines the thresholds required to consider a gene as over/under-expressed, in terms of inclusion of the gene expression value within the highest/lowest (n) percent of gene expression values; Gap, which defines the maximum number of non over/under-expressed genes allowed to be localized between two over/under-expressed genes in a cluster (if Gap = 0, only strictly contiguous genes will be considered to be in cluster); Gene Type, which defines if TRAM, while constructing the linear map of genes, will use only genes with an official gene symbol assigned, or will use also genes with at least an Entrez Gene identifier available, or will use any locus with at least a UniGene cluster identifier, even in absence of an official or Entrez Gene symbol.

The statistical significance of the results is calculated as described in the "Statistical analysis" Methods section below.

### Statistical analysis

To assess the statistical significance of the results, TRAM uses the hypergeometric distribution to test the probability 'P' that colocalization of over/under-expressed genes within the same chromosomal segment ('Map' analysis mode) or in the same cluster of contiguous genes ('Cluster' analysis mode) may be due to chance. To this aim, calculations are performed as previously described [10].

The 'P' value needs to be corrected to account for False Discovery Rate (FDR) due to the high number of segments or genes in a genome. The 'Q' field in TRAM displays the p-value corrected for FDR. Q (q-value) for each chromosomal segment or cluster of contiguous genes is defined as Q = (p*N)/i, where 'p' is the p-value of the segment or of the cluster, 'N' is the total number of segments or cluster considered (i.e., all those tagged as over/under-expressed under the criteria defined by the user selected analysis settings) and 'i' is the number of windows with a p-value not higher than 'p' [10]. Results are considered statistically significant for Q < 0.05.

Depending on the type of analysis selected by the user, TRAM may perform statistical significance computation taking into account all genes in the genome or, in order to emphasize local chromosomal effects, taking into account only the genes located in the same chromosome the chromosomal segment or gene cluster belongs to.

The results discussed above, regarding the outcome of the normalization methods implemented in TRAM, were obtained using descriptive statistical analysis functions of the software JMP 5 for Mac OS X (SAS Institute, Cary, NC).

### Biological model test

To test the software, we performed a meta-analysis of a dataset obtained by gene expression profiling of human hematopoietic progenitor cells, searching for up- or down-regulated chromosomal segments and gene clusters in human megakaryocyte (Mk) cells, the precursors of platelets, compared to CD34+ hematopoietic undifferentiated stem cells. After searching in the GEO database, we selected 9 human Mk samples and 19 human CD34+ cell samples, using the following criteria: homogeneity of cell type, derivation from different Authors works and representation of microarray platforms with different technology and number of spots (Table 1) [19-24]. The default analysis parameters were: expression values normalized both intra-sample (by percentage of sample mean) and inter-sample (by scaled quantile method); 2.5th percentile upper and lower threshold to define over- or under-expression, for both segments and genes, with respect to whole genome gene set; requirement of at least 3 over/under-expressed genes to define a segment accordingly; window (segment) of 500,000 bp (with shift of 250,000 bp). The wideness of the window and the minimum number of over/under-expressed genes required to lie in the window ('n') should be reciprocally adjusted so that the mean number of all genes included in a segment (shown at the end of the Segment Map) would exceed 'n'. In our human genes data set, setting a window to 500,000 bp led to segments containing a mean of 4.3 genes, while lowering the window to 250,000 bp led to a mean of 2.7 genes (segments with no gene value are ignored in the calculation of mean). In the 'Cluster' mode, all available genes including UniGene clusters of transcripts were selected to construct the map, and the Gap was set equal to 1. The test was run on March 2010, using Entrez Gene and UniGene data available at the time (UniGene build #222).

## Authors' contributions

SF, PS and LL conceived the software. PS and LL developed and tested the basic version of the software and wrote the software guide. PS conceived, built and analyzed the biological model used to test the software. FP and MG systematically tested the software, debugged the Windows version and revised and expanded the software guide. FFa, MCP, FFr and RC tested and improved the Macintosh version of the software and developed the organism-specific versions. LV tested the software and collaborated to the biological model test. SC tested the software and developed its graphical interface. SB and AC critically revised the software and the manuscript. GAD, GP and SF supervised the work and revised the whole manuscript. All authors drafted, critically discussed and approved the final manuscript as well as the software guide.
